# The FGF21 analog pegozafermin in severe hypertriglyceridemia: a randomized phase 2 trial

**DOI:** 10.1038/s41591-023-02427-z

**Published:** 2023-06-24

**Authors:** Deepak L. Bhatt, Harold E. Bays, Michael Miller, James E. Cain, Katarzyna Wasilewska, Nabil S. Andrawis, Teresa Parli, Shibao Feng, Lulu Sterling, Leo Tseng, Cynthia L. Hartsfield, Germaine D. Agollah, Hank Mansbach, John J. P. Kastelein, ENTRIGUE Principal Investigators

**Affiliations:** 1 Mount Sinai Heart, Icahn School of Medicine, Mount Sinai Health System, New York City, NY USA; 2 Louisville Metabolic and Atherosclerosis Research Center, University of Louisville School of Medicine, Louisville, KY USA; 3 Corporal Michael J. Crescenz VA Medical Center and Hospital of the University of Pennsylvania, Philadelphia, PA USA; 4 Family Medicine Clinic Science, Lampasas, TX USA; 5 ZDROWIE Osteo-Medic, Bialystok, Poland; 6 Manassas Clinical Research Center, Manassas, VA USA; 7 89bio Inc., San Francisco, CA USA; 8 Department of Vascular Medicine, Academic Medical Center, University of Amsterdam, Amsterdam, Netherlands

**Keywords:** Clinical trial design, Medical research

## Abstract

Pegozafermin, a long-acting glycopegylated analog of human fibroblast growth factor 21, is in development for the treatment of severe hypertriglyceridemia (SHTG) and nonalcoholic steatohepatitis. Here we report the results of a phase 2, double-blind, randomized, five-arm trial testing pegozafermin at four different doses (*n* = 67; 52 male) versus placebo (*n* = 18; 12 male) for 8 weeks in patients with SHTG (triglycerides (TGs), ≥500 mg dl^−1^ and ≤2,000 mg dl^−1^). Treated patients showed a significant reduction in median TGs for the pooled pegozafermin group versus placebo (57.3% versus 11.9%, difference versus placebo −43.7%, 95% confidence interval (CI): −57.1%, −30.3%; *P* < 0.001), meeting the primary endpoint of the trial. Reductions in median TGs ranged from 36.4% to 63.4% across all treatment arms and were consistent regardless of background lipid-lowering therapy. Results for secondary endpoints included significant decreases in mean apolipoprotein B and non-high-density lipoprotein cholesterol concentrations (−10.5% and −18.3% for pooled doses compared to 1.1% and −0.6% for placebo (95% CI: −21.5%, −2.0%; *P* = 0.019 and 95% CI: −30.7%, −5.1%; *P* = 0.007, respectively), as well as a significant decrease in liver fat fraction for pooled treatment (*n* = 17) versus placebo (*n* = 6; −42.2% pooled pegozafermin, −8.3% placebo; 95% CI: −60.9%, −8.7%; *P* = 0.012), as assessed in a magnetic resonance imaging sub-study. No serious adverse events were observed to be related to the study drug. If these results are confirmed in a phase 3 trial, pegozafermin could be a promising treatment for SHTG (ClinicalTrials.gov registration: NCT0441186).

## Abstract (web summary)

In a phase 2, randomized clinical trial in patients with severe hypertriglyceridemia, pegozafermin, a long-acting analog of human fibroblast growth factor 21, was safe and met the primary endpoint of the trial for reducing serum triglyceride levels.

## Main

Severe hypertriglyceridemia (SHTG; ≥500 mg dl^−1^) increases the risk for both acute pancreatitis and cardiovascular disease^1–10^. Although lifestyle modification strategies are commonly recommended as first-line treatment, triglyceride (TG) levels often remain elevated and require pharmacologic treatment in almost all patients^11–13^. Current therapies for SHTG rarely reduce TGs to desired levels, highlighting the need for new therapeutic options. Moreover, as SHTG is commonly associated with obesity, metabolic syndrome, insulin resistance, type 2 diabetes mellitus (T2DM) and nonalcoholic fatty liver disease (NAFLD)^12,14–16^, an ideal therapy should not only lower TG levels, but also provide benefit for other metabolic comorbidities.

Fibroblast growth factor 21 (FGF21) is an endogenous stress hormone that regulates lipid and glucose metabolism and energy expenditure. Preclinical data suggest that in the liver, FGF21 reduces fat via increased adenosine monophosphate-activated protein kinase (AMPK) signaling, which stimulates fatty acid oxidation and decreases de novo lipogenesis to mitigate new TG accumulation, and promotes TG secretion in the form of very-low-density lipoprotein (VLDL) to reduce existing fat stores. In adipose tissue, FGF21 improves insulin sensitivity and accelerates TG-rich lipoprotein turnover (for example, VLDL metabolism) as a result of activating brown adipose tissue and browning of white adipose tissue by inducing the expression of uncoupling protein 1 (refs. ^17–22^). Notably, FGF21 increases low-density lipoprotein receptor (LDLR) expression which could accelerate the uptake of the generated VLDL remnants via the ApoE-LDLR pathway^22^.

Pegozafermin is a glycopegylated recombinant analog of human FGF21 designed to have a longer half-life than native FGF21 while recapitulating the receptor activity profile of the native hormone. It is being developed for the treatment of SHTG and nonalcoholic steatohepatitis (NASH). Pegozafermin has previously demonstrated beneficial effects on serum lipids (TGs, low-density lipoprotein cholesterol (LDL-C), non-high-density lipoprotein cholesterol (non-HDL-C) and HDL-C), insulin resistance, HbA1c, body weight and liver fat in patients with NASH^23,24^. Although previous clinical trials with pegozafermin and other FGF21 analogs have consistently demonstrated improvements in lipids in both healthy volunteers and patients with NASH or diabetes, FGF21 analogs have not been assessed in SHTG^23,25–29^. To the best of our knowledge, ENTRIGUE was the first clinical trial to investigate an FGF21 analog as a new therapeutic agent for the treatment of SHTG.

## Results

### Patient characteristics

**Fig. 1:**
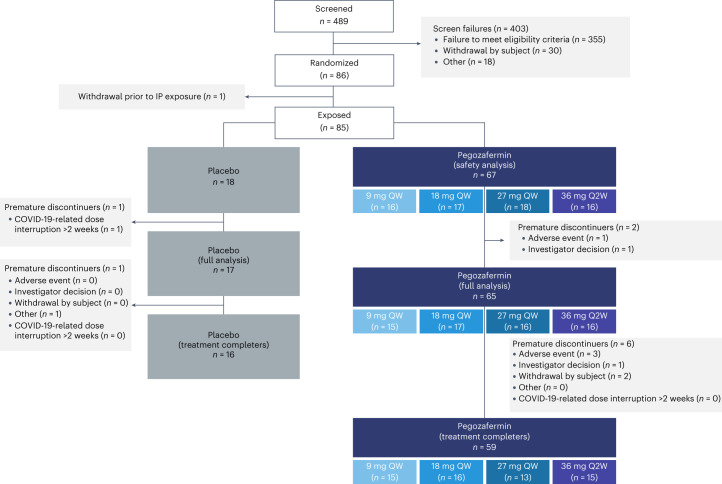
Patient flow diagram. CONSORT flow diagram showing participant disposition and data analysis sets, including reasons for discontinuation. Of the 85 participants treated, 75 (88.2%; *n* = 59 in pegozafermin and *n* = 16 in placebo) completed treatment. QW, once-weekly; Q2W, once every 2 weeks.

From November 2020 to February 2022, 489 patients underwent screening, with 85 (17.4%) patients randomized and treated. Among the patients treated with pegozafermin, the distribution was as follows: placebo (*n* = 18); 9 mg once weekly (QW, *n* = 16); 18 mg QW (*n* = 17); 27 mg QW (*n* = 18); and 36 mg every 2 weeks (Q2W, *n* = 16). Postbaseline TG levels were available for the 82 patients in the full analysis set. The baseline characteristics of the patients, shown in Table 1, were reasonably balanced across groups, with a mean age of 53.7 years, 75.3% male, mean body mass index (BMI) 33.1 kg m^−^^2^, 50.6% with T2DM, 55.3% on background lipid-lowering therapy (including statins, prescription omega-3 fatty acids, fibrates (fibrate cohort), bempedoic acid and ezetimibe) and a median baseline TG level of 622.0 mg dl^−1^. Other baseline lipids were at typical mean levels for this population as follows: LDL-C (89.1 mg dl^−1^); HDL-C (28.4 mg dl^−1^); and non-HDL-C (211.5 mg dl^−1^). At clinical sites with magnetic resonance imagining (MRI) capability, a subset of patients (*n* = 24) underwent baseline proton density fat fraction (PDFF) evaluation to measure hepatic steatosis. All patients assessed by MRI-PDFF had evidence of fatty liver (>5% hepatic fat) at baseline, with an overall mean value of 20.1% (Table 1). Patient disposition and population analysis sets are presented in Fig. 1 and Extended Data Table 1, respectively.

**Table 1:** Demographics and baseline characteristics

Characteristic, mean or %	Placebo (*n* = 18)	PGZ pooled (*n* = 67)	PGZ 9 mg QW (*n* = 16)	PGZ 18 mg QW (*n* = 17)	PGZ 27 mg QW (*n* = 18)	PGZ 36 mg Q2W (*n* = 16)	Total (*n* = 85)
Age, years	57.5	52.7	54.6	49.2	53.9	53.1	53.7
Male, %	66.7	77.6	68.8	82.4	72.2	87.5	75.3
White, %	94.4	95.5	93.8	100	100	87.5	95.3
BMI, kg m^−^^2^	33.1	33.1	32.9	32.3	34.2	32.9	33.1
Type 2 diabetes, *n* (%)	11 (61.1)	32 (47.8)	9 (56.3)	6 (35.3)	10 (55.6)	7 (43.8)	43 (50.6)
Hypertension, *n* (%)	13 (72.2)	39 (58.2)	11 (68.8)	7 (41.2)	11 (61.1)	10 (62.5)	52 (61.2)
Triglyceride, mg dl^−1^(median)	574.8	631.3	593.3	633.3	645.3	688.5	622.0
Triglyceride <750 mg dl^−1^at screening, %	66.7	59.7	62.5	58.8	61.1	56.3	61.2
Triglyceride ≥750 mg dl^−1^at screening, %	33.3	40.3	37.5	41.2	38.9	43.8	38.8
Non-HDL-C, mg d^l−1^	219.6	209.3	216.2	203.2	203.4	215.4	211.5
HDL-C, mg dl^−1^	28.3	28.4	30.7	27.3	30.6	24.8	28.4
LDL-C, mg dl^−1^	87.9	89.4	91.6	88.3	97.3	79.5	89.1
VLDL-C, mg dl^−1^	133.2	117.8	123.2	115.0	104.7	130.1	120.9
VLDL triglyceride, mg dl^−1^	610.2	633.6	588.0	574.2	590.0	791.4	628.9
Total cholesterol, mg dl^−1^	247.9	237.6	246.9	230.5	234.0	240.1	239.8
Apolipoprotein B, mg dl^−1^	116.3	115.3	120.1	115.3	119.3	105.9	115.5
Apolipoprotein C3, mg dl^−1^	29.7	29.5	29.4	28.0	30.7	30.0	29.6
Apolipoprotein A1, mg dl^−1^	138.8	137.1	143.3	137.7	141.0	125.9	137.5
Lipoprotein (a), nmol l^−1^	42.5	45.4	48.2	21.1	55.1	58.3	44.8
Free fatty acids, mmol l^−1^	0.6	0.5	0.5	0.6	0.6	0.5	0.5
HbA1c, %	6.28	6.55	6.63	6.59	6.61	6.37	6.50
HbA1c ≥ 6.5%, *n* (%)	7 (38.9)	30 (44.8)	9 (56.3)	6 (35.3)	9 (50.0)	6 (37.5)	37 (43.5)
High-sensitivity C-reactive protein, mg l^−1^	4.6	4.5	5.9	3.6	3.2	5.7	4.6
Adiponectin, µg ml^−1^	4.0	3.3	3.3	2.4	4.9	2.5	3.5
Fasting plasma glucose, mg dl^−1^	124.4	148.7	158.5	139.3	157.5	139.0	143.6
ALT, U l^−1^	29.1	33.9	36.3	36.9	33.0	29.2	32.8
AST, U l^−1^	24.2	24.7	26.7	27.6	23.7	20.6	24.6
Background lipid-modifying therapy, *n* (%)							
Any	11 (61.1)	36 (53.7)	8 (50.0)	9 (52.9)	11 (61.1)	8 (50.0)	47 (55.3)
Statins	9 (50.0)	29 (43.3)	6 (37.5)	9 (52.9)	7 (38.9)	7 (43.8)	38 (44.7)
High-intensity statins	4 (22.2)	17 (25.4)	6 (37.5)	5 (29.4)	4 (22.2)	2 (12.5)	21 (24.7)
Prescription fish oils	2 (11.1)	10 (14.9)	1 (6.3)	2 (11.8)	4 (22.2)	3 (18.8)	12 (14.1)
Fibrates	3 (16.7)	3 (4.5)	0	0	3 (16.7)	0	6 (7.1)
Ezetimibe	1 (5.6)	8 (11.9)	2 (12.5)	2 (11.8)	2 (11.1)	2 (12.5)	9 (10.6)
Bempedoic acid	0	1 (1.5)	0	1 (5.9)	0	0	1 (1.2)
Liver fat fraction by MRI-PDFF, % (*n* = 24)	16.5 (*n* = 6)	21.3 (*n* = 18)	19.8 (*n* = 3)	18.0 (*n* = 5)	22.4 (*n* = 7)	25.5 (*n* = 3)	20.1 (*n* = 24)

ALT, alanine aminotransferase; AST, aspartate aminotransferase; BMI, body mass index; HbA1c, glycated hemoglobin; HDL, high-density lipoprotein; LDL, low-density lipoprotein; MRI-PDFF, magnetic resonance imaging proton density fat fraction; PGZ, pegozafermin; QW, once weekly; Q2W, once every 2 weeks; VLDL, very-low-density lipoprotein.

### Efficacy endpoints

#### Effect on triglyceride levels (primary endpoint)

**Fig. 2:**
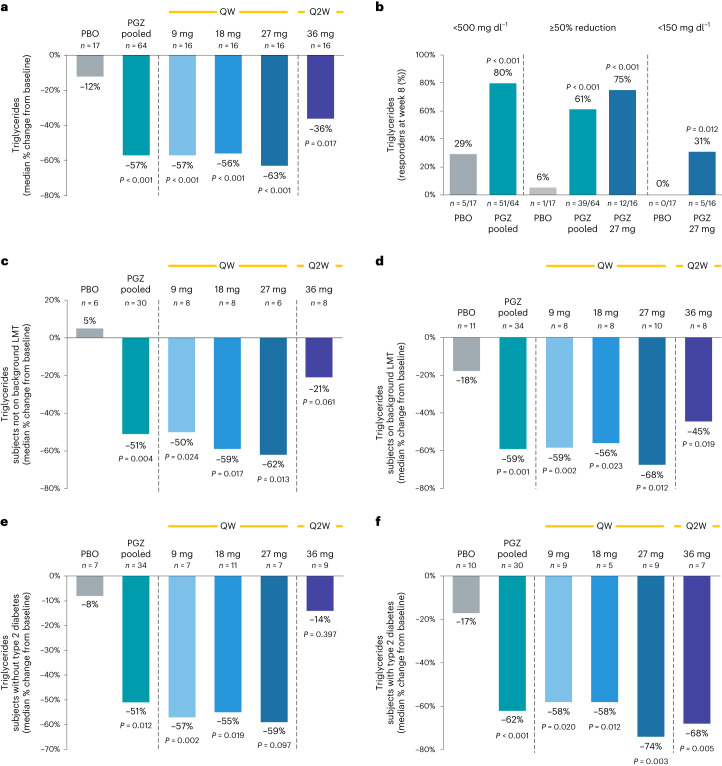
Effect of pegozafermin on serum TGs. **a**, Median percent change in TGs from baseline to week 8 (the primary endpoint). **b**, Proportion of participants who achieved TG responses of <500 mg dl^−1^, <150 mg dl−1 or a ≥ 50% reduction from baseline to week 8. **c**–**f**, TG subgroup analysis among participants (not on background LMT (**c**), on background LMT (**d**), without T2DM (**e**) and with T2DM (**f**). Data are based on the full analysis set population (defined as all randomized participants who received at least one dose of study treatment, had baseline and at least one postbaseline TG value) and analyzed using the van Elteren test for pooled pegozafermin groups and the Wilcoxon rank-sum test for individual pegozafermin dose groups. *n* represents independent participants examined at baseline and four postbaseline timepoints for TG-related graphs. All *P* values are two-sided and based on comparison to the placebo arm. PBO, placebo; PGZ, pegozafermin; QW, once weekly; Q2W, once every two weeks.

Pegozafermin substantially reduced TGs after 8 weeks of therapy across all dose groups, with placebo-corrected changes from baseline ranging from −29.0% to −52.9%. Pooled pegozafermin data showed a placebo-corrected median percent change in TG levels of −43.7% (−57.3% versus −11.9% placebo; 95% confidence interval (CI): −57.1%, −30.3%; *P* < 0.001; Fig. 2a). A total of 79.7% of patients treated with pegozafermin achieved a target TG level of <500 mg dl^−1^, compared with 29.4% of patients on placebo (52.1% placebo-corrected, 95% CI: 29.4%, 74.7%; *P* < 0.001; Fig. 2b). Furthermore, 60.9% of all patients treated with pegozafermin had reductions of ≥50% from baseline, compared with 5.9% of patients on placebo (53.1% placebo-corrected, 95% CI: 36.7%, 69.5%; *P* < 0.001), while at the highest QW dose (27 mg), 75.0% of patients had a TG reduction of ≥50% from baseline (69.1% placebo-corrected, 95% CI: 45.1%, 93.1%; *P* < 0.001) and 31.3% were able to normalize their TG to <150 mg dl^−1^ compared with 0% of patients on placebo (31.3% placebo-corrected, 95% CI: 8.5%, 54.0%; *P* = 0.012; Fig. 2b). TG reduction was comparable across all prespecified groups (Extended Data Fig. 2) and remained consistent irrespective of background lipid-lowering therapy or T2DM status (Fig. 2c–f).

#### Effects on overall lipid profile

**Fig. 3:**
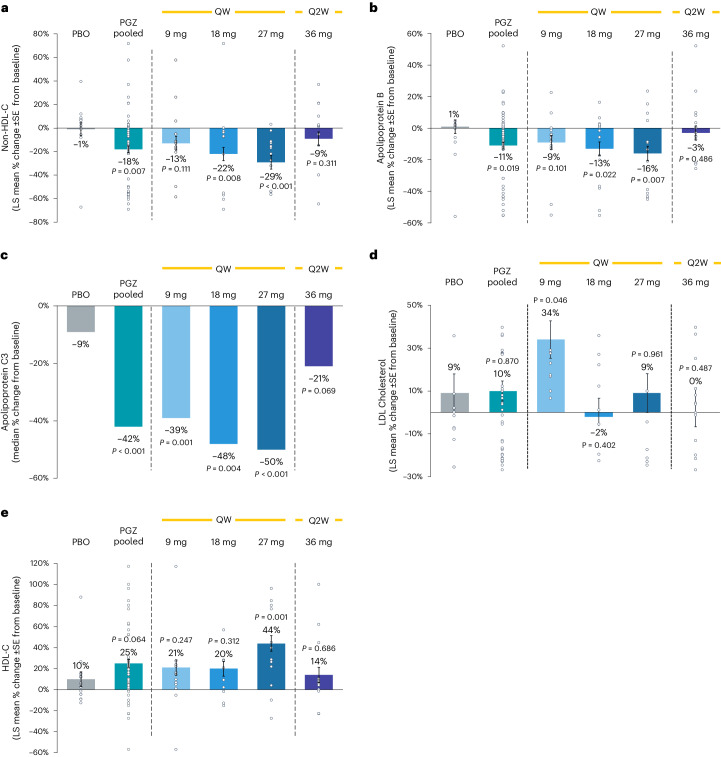
Effect of pegozafermin on serum lipids. **a**–**e**, LS mean (±s.e.) or median (ApoC3) percent change in non-HDL-C (**a**), apolipoprotein B (**b**), apolipoprotein C3 (**c**), LDL-C (**d**) and HDL-C (**e**) from baseline to week 8. Data are based on the full analysis set population (defined as all randomized participants who received at least one dose of study treatment, had baseline and at least one postbaseline TG) and analyzed via MMRM. *n* represents independent participants examined at baseline and two postbaseline timepoints for lipid-related graphs. All *P* values are two-sided and based on comparison to the placebo arm. PBO, placebo; PGZ, pegozafermin; QW, once weekly; Q2W, once every two weeks.

Treatment with pegozafermin resulted in improvements in non-HDL-C and apolipoprotein B (ApoB), with least squares (LS) mean percent changes from baseline for pooled pegozafermin of −18.3% versus −0.6% for placebo (−17.9% placebo-corrected, 95% CI: −30.7%, −5.1%; *P* = 0.007) and −10.5% versus 1.1 % for placebo (−11.8% placebo-corrected, 95% CI: −21.5%, −2.0%; *P* = 0.019), respectively (Fig. 3a,b). Treatment with pegozafermin also led to a significant reduction in ApoC3 (median percent change –41.9% versus –8.9% placebo (–32.0% placebo-corrected, 95% CI: −44.7%, −18.0%; *P* < 0.001); Fig. 3c). Although minimal changes in LDL-C were detected in pooled pegozafermin (Fig. 3d), LS mean percent change in HDL-C levels from baseline in participants treated with pegozafermin receiving the 27 mg weekly dose significantly increased (44.5% versus 9.7% for placebo (34.8% placebo-corrected, 95% CI: 14.5%, 55.1%; *P* = 0.001); Fig. 3e). Treatment with pegozafermin 27 mg weekly also resulted in a 73% decrease in ApoB48, suggesting improved clearance of plasma chylomicrons and their remnants, in addition to reductions in ApoB100 particles. Additional lipid data are available in Extended Data Table 2.

#### Hepatic and metabolic effects

**Fig. 4:**
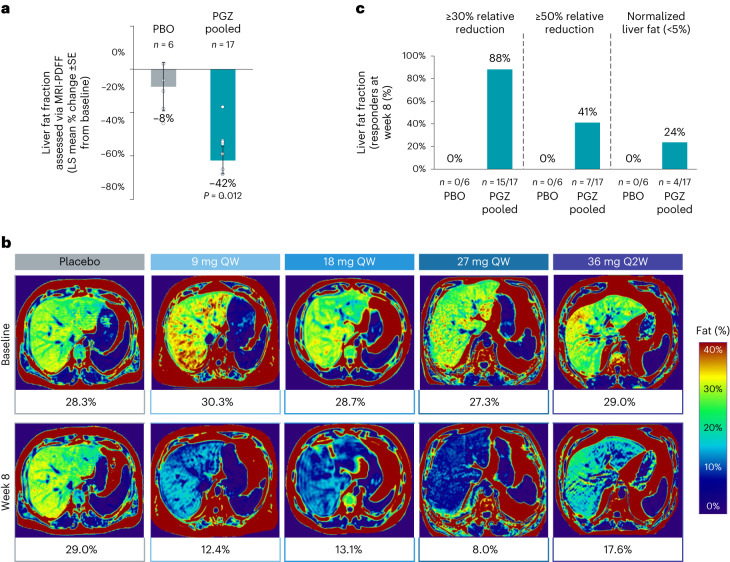
Effect of pegozafermin on hepatic steatosis. **a**, LS mean (±s.e.) percent change from baseline to week 8 in liver fat fraction assessed by MRI-PDFF. **b**, MRI-PDFF images depicting changes in liver fat fraction from representative participants with elevated baseline liver fat fraction defined as >25%. **c**, Proportion of participants who achieved liver fat normalization (that is, <5% by MRI-PDFF), ≥30% or ≥50% relative reduction in liver fat after 8 weeks. Data are based on full analysis set population (defined as all randomized participants who received at least one dose of study treatment, had baseline and at least one postbaseline TG) and analyzed using MMRM or the van Elteren test for pooled pegozafermin groups and Wilcoxon rank-sum test for individual pegozafermin dose groups. *n* represents independent participants examined at baseline and one postbaseline timepoint for liver fat graphs. All individual MRI-PDFF images in **b** were generated as 384 × 288 mm and color corrected to a common color scale to allow direct comparison across images. MRI-PDFF, magnetic resonance imaging whole liver proton density fat fraction; PBO, placebo; PGZ, pegozafermin, QW, once weekly; Q2W, once every two weeks.

Patients treated with pegozafermin for 8 weeks had significant reductions in liver steatosis compared with placebo (LS mean percent change −42.2% versus −8.3%; 95% CI: −60.9%, −8.7%; *P* = 0.012; Fig. 4a). Representative MRI-PDFF images are shown in Fig. 4b, with all individual treatment images and responses presented in Extended Data Figs. 3 and 4. Many patients treated with pegozafermin attained important clinical thresholds, including ≥30% reduction, ≥50% reduction or normalization of liver fat (defined as <5%), with response rates of 88%, 41% and 24%, respectively, compared with 0% in placebo across all measurements (Fig. 4c). Patients receiving the 27 mg weekly dose also had improvement in inflammatory markers as follows: alanine aminotransferase, aspartate aminotransferase and high-sensitivity c-reactive protein (Extended Data Table 3).

### Safety

Treatment-emergent adverse events (TEAEs) were reported in 41/67 (61.2%) of patients treated with pegozafermin versus 9/18 (50%) on placebo (Table 2). The most common TEAEs were related to gastrointestinal disturbances and injection site reactions, all of which were mild to moderate, with the majority transient in duration. In the pooled pegozafermin group, nausea, diarrhea and injection site reactions occurred at rates of 13.4%, 10.4% and 9%, respectively, compared with 0%, 5.6% and 0% for placebo. The percent of TEAEs was higher for nausea (27.8%) and diarrhea (22.2%) in the 27 mg weekly dose. No grade 3 or higher TEAEs were reported. There was no difference in clinically significant shifts for blood pressure between placebo and pegozafermin treatment groups at week 8. The mean change in systolic pressure for placebo and pegozafermin was −4.1 mmHg and 0.7 mmHg at week 4 and 0.3 mmHg and 1.7 mmHg at week 8, respectively. However, one serious TEAE of hypertension was reported in the 27 mg QW arm in a patient with newly diagnosed hypertension before enrollment, which was deemed unrelated to treatment and led to study discontinuation. There were three additional treatment-emergent discontinuations in the 27 mg arm as follows: two patients with TEAEs considered related by the investigator (one with nausea and vomiting and one with abdominal cramps) and one patient with nausea and abdominal pain assessed as unrelated to pegozafermin (Table 2). No deaths, systemic hypersensitivity reactions or adverse events of liver transaminase elevation were reported.

**Table 2:** Summary of safety and tolerability

	Placebo (*n* = 18)	PGZ pooled (*n* = 67)	PGZ 9 mg QW (*n* = 12)	PGZ 18 mg QW (*n* = 21)	PGZ 27 mg QW (*n* = 18)	PGZ 36 mg Q2W (*n* = 16)
TEAEs	9 (50.0)	41 (61.2)	7 (58.3)	13 (61.9)	14 (77.8)	7 (43.8)
Grade 1 (mild)	5 (27.8)	22 (32.8)	6 (50.0)	7 (33.3)	6 (33.3)	3 (18.8)
Grade 2 (moderate)	4 (22.2)	19 (28.4)	1 (8.3)	6 (28.6)	8 (44.4)	4 (25.0)
Grade >3 (severe)	0	0	0	0	0	0
Serious TEAEs	0	1 (1.5)	0	0	1 (5.6)	0
Hypertension	0	1 (1.5)	0	0	1 (5.6)	0
TEAEs related to treatment	2 (11.1)	23 (34.3)	5 (41.7)	6 (28.6)	7 (38.9)	5 (31.3)
Serious TEAEs related to treatment	0	0	0	0	0	0
TEAEs leading to treatment discontinuation	0	4 (6.0)	0	0	4 (22.2)	0
Hypertension	0	1 (1.5)	0	0	1 (5.6)	0
Abdominal pain	0	2 (3.0)	0	0	2 (11.1)	0
Nausea	0	2 (3.0)	0	0	2 (11.1)	0
Vomiting	0	1 (1.5)	0	0	1 (5.6)	0
TEAEs reported by ≥5% in pooled PGZ groups						
Nausea	0	9 (13.4)	1 (8.3)	1 (4.8)	5 (27.8)	2 (12.5)
Diarrhea	1 (5.6)	7 (10.4)	2 (16.7)	1 (4.8)	4 (22.2)	0
Injection site reaction	0	6 (9.0)	1 (8.3)	2 (9.5)	1 (5.6)	2 (12.5)
COVID-19	3 (16.7)	4 (6.0)	0	3 (14.3)	0	1 (6.3)
Injection site erythema	0	4 (6.0)	0	1 (4.8)	2 (11.1)	1 (6.3)
Injection site pruritus	0	4 (6.0)	1 (8.3)	2 (9.5)	1 (5.6)	0
Abdominal pain	0	3 (4.5)	0	0	2 (11.1)	1 (6.3)

COVID-19, coronavirus disease-19; TEAE, treatment-emergent adverse event; PGZ, pegozafermin.

Safety analysis set is defined as all participants who received at least one dose of investigational product. The safety analysis set is summarized based on planned treatment. Four participants randomized to receive 9 mg QW received 18 mg QW throughout the treatment; these four participants were categorized in the actual treatment group of 18 mg QW in safety analysis. Three participants randomized to receive 27 mg QW received 36 mg QW throughout the treatment; these three participants were categorized in the planned treatment group of 27 mg QW as it was the highest QW dose in the study.

## Discussion

This placebo-controlled, randomized study demonstrated that treatment with the FGF21 analog pegozafermin resulted in significant reductions in TGs in patients with SHTG. Significant reductions were also observed in atherogenic lipoproteins, including non-HDL-C and ApoB, as well as ApoC3, an important regulator of lipoprotein lipase, suggesting pegozafermin reduces production and improves clearance of TG-rich lipoproteins. Whereas the levels of LDL-C remained relatively stable, there was a numerical increase in HDL-C across all doses, most notably at the 27-mg dose; the 27-mg dose was also the most efficacious for reducing TGs and lipoproteins. Every-other-week dosing had less impact across the various outcome parameters, likely due to the pharmacokinetics of the drug and volatility of TG levels.

Eligibility criteria allowed for the enrollment of participants on stabilized regimens of approved lipid-modifying therapies (LMTs), such as statins, prescription fish oil and/or fibrates. Approximately 55% of participants enrolled were on background LMT, with a majority on a statin (45% (25% high-intensity statin)), followed by prescription fish oil (14%) and fibrates (7%). Initially, fibrates had been excluded due to potential crosstalk between FGF21 and peroxisome proliferator-activated receptor alpha (PPARα) pathways^30^, but the study was ultimately amended to include a fibrate cohort, with the additional criteria that those participants must have had at least 6% liver fat at baseline. Enrollment into this arm proved difficult (*n* = 6) and, therefore, participants on fibrates were likely underrepresented in the final study population relative to clinical practice. Nonetheless, with the exception of this potential caveat regarding fibrates, the overall utilization of LMTs in the study population appeared to generally reflect real-world treatment patterns in patients with SHTG. Christian et al. reported that baseline medication use (up to 6 months preceding the index date) was approximately 31% for statins and 14% for TG-lowering medications^31^. In the same study, follow-up medication use after the index date slightly increased to 38% and 35% for statins and TG-lowering medications, respectively, leaving a substantial number of patients still untreated. Similarly, a study by Toth et al. reported that 30–50% of TG-treatment-naive patients had not initiated any pharmacotherapy within 4 months of their index date (of those who were prescribed medication, approximately 50% received a statin, 30% received fibrates and 8% received omega-3 fatty acids)^32^. More recently, data from the Rochester Epidemiology Project showed that only 46% of patients with primary isolated hypertriglyceridemia (TG ≥ 500 mg dl^−1^) were on LMTs within 18 months after the detection of elevated TG levels^33^.

In the current study, it should be noted that the effect of pegozafermin remained consistent regardless of the presence or absence of background LMT, suggesting that pegozafermin can substantially improve many important lipid parameters when used as an adjunct to diet and exercise or as add-on therapy in patients who are unable to achieve TG < 500 mg dl^−1^. Furthermore, the effect of pegozafermin was comparable irrespective of LMT drug classification. This is an important finding, as data in patients with residual dyslipidemia on LMT in the United States showed that only 36.5% of such patients were at goal or near normal levels for TG, LDL-C and HDL-C^34^.

While the primary target for CV risk reduction remains LDL-C, identifying new therapies that can address residual CV risk after LDL treatment is of significant interest. Most clinical trials using therapies that lower TGs (that is, fibrates, niacin and omega-3 fatty acids, with the exception of icosapent ethyl (IPE)) have not demonstrated an add-on reduction in CV events in patients on statin therapy^4^. For instance, while the REDUCE-IT trial (IPE, 4 g per day) did find a 25% reduction (*P* < 0.001) in a composite of CV events in high-risk patients, only part of the benefit was predicted by TG lowering, suggesting the outcomes were attributable to broader pleiotropic effects of IPE^9^. The PROMINENT trial found no benefit on CV outcomes, despite TG reductions with pemafibrate^35^. The aforementioned trials were conducted in patients with TG < 500 mg dl^−1^, so extrapolation to an SHTG population requires caution. PROMINENT reported that pemafibrate demonstrated a modest decrease in TG (−26.2%) and VLDL-C (−25.8%), but led to placebo-corrected increases in LDL-C, ApoB and non-HDL-C (10, 5 and 3 mg dl^−1^, respectively) with no differences in CV outcomes^35^. Similar to pemafibrate, an increase in LDL-C was also observed in the recently published paper on evinacumab (an angiopoietin-like 3 inhibitor (ANGPTL3)) in patients with SHTG across three cohorts with and without lipoprotein lipase pathway mutations, although it should be noted that both non-HDL-C and ApoB were decreased in this study^36^. It is not unexpected that LDL-C may increase, sometimes dramatically, in patients being treated for SHTG, particularly with agents such as fenofibrate or EPA/DHA. This is known as the ‘beta-shift’ phenomenon, where LDL-C levels can rise due to increased lipolysis of VLDL via lipoprotein lipase^37^. In the current study, there was a relatively small increase in LDL-C that did not differ from placebo; however, one might speculate that 45% of patients receiving background statin therapy could have impacted the observed LDL-C response. Indeed, a post hoc analysis of ENTRIGUE patients on background LMT (which included but was not limited to statin therapy) demonstrated a placebo-corrected LS mean difference in LDL-C of −9.0%, compared with an LS mean difference of 1.7% for pooled pegozafermin groups^38^. Patients enrolled in this trial had a mean baseline LDL-C of <90 mg dl^−1^, which is relatively well-controlled for this population, so any impact related to the minimal change in LDL-C is likely to be offset by the significant decreases in both non-HDL-C and ApoB, as well as meaningful reductions in TG-rich lipoprotein cholesterol (TRL-C; Extended Data Table 2), when considering the overall atherogenic burden.

Newer RNA-based therapies (antisense oligonucleotide (ASO) and RNA interference (RNAi)) also are being developed to substantially lower TG in both familial chylomicronemia syndrome (FCS) and SHTG. Early data suggest that traditional ASO approaches may be associated with safety concerns. For example, while volanesorsen (ASO *APOC3* inhibitor) is approved in the EU for the treatment of FCS^39^, it has not gained FDA approval because of concerns regarding bleeding and thrombocytopenia. In addition, Pfizer and Ionis discontinued their vupanorsen clinical program (ASO targeting *ANGPTL3*) because of the modest effects on non-HDL-C and TG reduction and association with dose-dependent increases in liver fat and liver enzymes^40^. The development of second-generation ASOs, such as olezarsen (*APOC3* inhibitor), has been a major advancement, which is reflected in its FDA fast-track designation for patients with FCS. siRNA agents also appear promising, although some safety signals appear to be associated with these agents as well. Data from the SHASTA-2 trial evaluating ARO*-APOC3* for SHTG suggest that this agent may be associated with increases in LDL-C, although data from the ARCHES-2 trial for mixed dyslipidemia evaluating ARO*-ANGPTL3* demonstrated a reduction in LDL-C^41,42^. Whether the differential effects on LDL-C are related to the disparate baseline TG levels in the two populations or dependent on the difference in gene targets remains unclear. Interestingly, both trials reported increased HbA1c in the treatment arm, particularly in patients with baseline diabetes.

In the 8-week study reported here, pegozafermin substantially reduced TG, non-HDL-C, ApoB and liver fat; increased HDL-C with minimal change in LDL-C; and improved liver transaminases, all while maintaining a favorable safety and tolerability profile. Taken together, these data suggest that pegozafermin provides an overall metabolic benefit with, as yet, no identified safety signals.

While patients with SHTG have an elevated risk for CV events, the primary clinical risk in patients with TG ≥ 500 mg dl^−1^ is acute pancreatitis, owing to saturation of, or impairment in, lipoprotein lipase-mediated lipolysis. The consequence is the accumulation of TG-rich particles that are hydrolyzed by pancreatic lipase, release of free fatty acids and subsequent pro-inflammatory signaling in adjacent pancreatic tissues^43^. Preclinical data suggest that FGF21 may have a role in modulating the inflammation and damage induced by experimental pancreatitis^44^. Furthermore, FGF21 has been postulated to promote β-cell survival and to protect isolated rat islets and insulin-producing INS cells from glucolipotoxicity and cytokine-induced apoptosis^44^.

Valdivielso et al. demonstrated that elevated levels of chylomicrons are necessary to trigger acute pancreatitis in the setting of high serum TGs^45^. Participants in the current study had markedly elevated ApoB48, a specific marker of chylomicron particles, at baseline (median range of 2.60–4.90 mg dl^−1^) compared with healthy participants (median, 0.51 mg dl^−1^), hyperlipidemic participants (median, 0.7 mg dl^−1^) and participants with obesity (median, 0.82 mg dl^−1^), putting them at an increased risk for developing acute pancreatitis^46^. Pegozafermin reduced ApoB48 robustly (73% reduction for the 27-mg weekly dose; Extended Data Table 2), suggesting an ability to improve clearance of chylomicrons and chylomicron remnants. Recently, Taskinen et al. demonstrated that patients with loss-of-function mutations in *APOC3*, which increases lipoprotein lipase activity, had lower plasma concentrations of VLDL, IDL and ApoB48 particles^47^. CM-ApoB48 and VLDL ApoB100 production rates were not affected, indicating that enhanced remnant removal may be the predominant mechanism for the observed reduction.

Pegozafermin demonstrated a robust 50% reduction of ApoC3 at the 27-mg dose, suggesting that increased lipoprotein lipase activity may contribute to the observed ApoB48 reduction. Indeed, post hoc analyses assessing the correlation between percent change in TG and ApoC3 in the pooled pegozafermin group at week 8 demonstrated a reasonable correlation between the two (Pearson *r* (linear correlation) = 0.87; Spearman *r* = 0.80, with *P* < 0.001), which indicates that greater reductions in TG were accompanied by greater reductions in ApoC3.

Current guidelines from the National Cholesterol Education Program Adult Treatment Panel III recommend reducing TGs to <500 mg dl^−1^ to prevent acute pancreatitis, with a secondary focus on decreasing CV risk. Data from a large retrospective claims study have demonstrated a lower incidence of clinical events for patients with SHTG who had follow-up TG levels of <400 mg dl^−1^, with significant incidence rate ratios in patients with follow-up TGs of <300 mg dl^−1^ for pancreatitis, overall CV events, acute myocardial infarction, heart failure, revascularization and acute coronary syndrome. However, the greatest clinical benefit (overall more robust incidence rate ratios and additional significance in ischemic stroke) was seen when follow-up levels were driven below 200 mg dl^−1^ (ref. ^31^). In the current trial, 80% of patients receiving pegozafermin (pooled data) were able to drive their TG below 500 mg dl^−1^, with 44% and 31% of participants receiving the 27-mg weekly dose achieving TG levels of <200 mg dl^−1^ and <150 mg dl^−1^, respectively, suggesting that pegozafermin may favorably impact the risk of acute pancreatitis and CV events.

Patients with SHTG often have metabolic comorbidities associated with dyslipidemia and insulin resistance, such as obesity, metabolic syndrome, T2DM and NAFLD, further increasing the risk of cardiovascular morbidity and mortality. Dramatic increases in obesity and T2DM over the past decades have exacerbated the development of NAFLD, making it a rising health concern in the United States and globally. NAFLD is currently the most common form of chronic liver disease in the United States and is often considered the hepatic manifestation of metabolic syndrome, a patient population that frequently suffers from atherogenic dyslipidemia. An important finding of the present study was the prevalence of liver fat in this population with SHTG—100% of patients who underwent MRI-PDFF screening had baseline hepatic steatosis, as defined by >5% liver fat (range 6.2–39.2%). Interestingly, baseline MRI-PDFF values did not correlate with baseline TG values, although every patient tested who had a baseline fasting TG level of >500 mg dl^−1^ had greater than 5% hepatic steatosis.

Pegozafermin therapy demonstrated significant reductions in fat accumulation in the liver, hitting key reduction targets of ≥30% and ≥50% in 88% and 41% of participants, respectively. These are important thresholds as it has been established in the literature that a ≥30% relative reduction in MRI-PDFF is associated with histologic response (categorized as a responder) and that a ≥50% relative reduction in MRI-PDFF evokes a substantially higher histologic response (defined as a super responder)^48^. In addition to the strong association of hepatic steatosis and histology, the presence of fatty liver has also been associated with more severe acute pancreatitis which can lead to a higher incidence of local complications, persistent organ failure and mortality regardless of underlying etiology^49,50^. More recently, Wu et al. reported that hyperlipidemia pancreatitis had the highest incidence of NAFLD (65%) and that the severity of acute pancreatitis, incidence of systemic inflammatory response syndrome and organ failure were higher in patients with NAFLD versus a non-NAFLD group^51^.

Pegozafermin treatment was able to normalize liver fat to ≤5% in 24% of participants in just 8 weeks. The data presented here report a significant reduction in quantified liver fat with a treatment targeting TG-rich lipoproteins in SHTG and suggest a potential benefit for lowering the risk of severe acute pancreatitis. The mechanism by which pegozafermin lowers liver fat remains to be fully characterized. Based on preclinical data in hepatocytes, FGF21 is thought to affect hepatic steatosis by modulating AMPK phosphorylation to regulate lipid accumulation, reducing sterol regulatory element-binding transcription factor 1 (SREBF1) to inhibit lipid synthesis, increasing *PPARα* mRNA and PPARα translocation into the nucleus to impact fatty acid oxidation and promoting lipid transport and secretion of VLDL^21^. In addition, FGF21 appears to increase hepatic expression of LDLR, which functions not only to clear VLDL and LDL from the circulatory system but also to promote the post-translational degradation of ApoB to subsequently reduce secretion of VLDL particles^21^. In adipose tissue, FGF21 accelerates TRL turnover as a result of activating brown adipose tissue and browning of white adipose tissue^22,52^. In addition, FGF21 has been shown to suppress adipose tissue lipolysis, increase adiponectin levels and decrease insulin resistance which also may impact hepatic steatosis^18,53^. Overall, the safety and tolerability profiles of pegozafermin were consistent with previous data, with mild-to-moderate gastrointestinal disturbance being the most common TEAEs^23,24^. There were no serious TEAEs related to the study drug. One limitation of this study is the lack of power to assess clinical events, such as pancreatitis, liver failure or cardiovascular endpoints. Another is that the majority of participants were White men, which may limit the generalizability of the data. While we acknowledge that fibrate use was likely underrepresented in the study, our overall utilization patterns are very similar to other reported real-world data in patients with SHTG. Further safety and tolerability data from a longer period of drug exposure at the target dose are necessary.

In summary, the FGF21 analog pegozafermin substantially reduced atherogenic lipoproteins, ApoC3 and liver fat in patients with SHTG and has the potential to positively impact other aspects of metabolic dysregulation. Indeed, these ‘metabolic patients’ are likely to benefit the most from a therapy that can function as a metabolic regulator across multiple comorbidities. If these findings are confirmed in an appropriately powered phase 3 trial, pegozafermin may be useful to treat SHTG and simultaneously address several other cardiometabolic risk factors.

## Methods

### Trial design

The ENTRIGUE trial was a randomized, double-blind, placebo-controlled, dose-ranging, phase 2 trial designed to assess the efficacy, safety, and tolerability of pegozafermin administered subcutaneously QW or Q2W in participants with SHTG. Participants with screening fasting TG ≥ 500 mg dl^−1^ (5.6 mmol l^−1^) and ≤2,000 mg dl^−1^ (22.6 mmol l^−1^) were eligible to enroll regardless of background LMT of statins, prescription omega-3 fatty acids and fibrates (fibrate expansion cohort only). The sex of participants, which was determined by self-report, was not considered in the study design.

**Extended Data Fig. 1:**
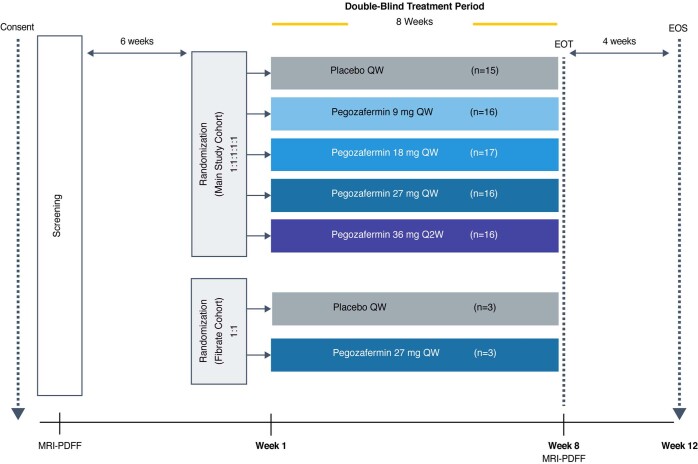
Study design. Study design showing the main study and fibrate cohorts as randomized. The 6-week screening period consisted of a 4-week medication/lifestyle stabilization period followed by a 1- to 2-week TG qualification period. EOS, end of study; EOT, end of treatment; MRI-PDFF, magnetic resonance imaging whole liver proton density fat fraction; TG, triglycerides; QW, once weekly; Q2W, once every 2 weeks.

**Extended Data Fig. 2:**
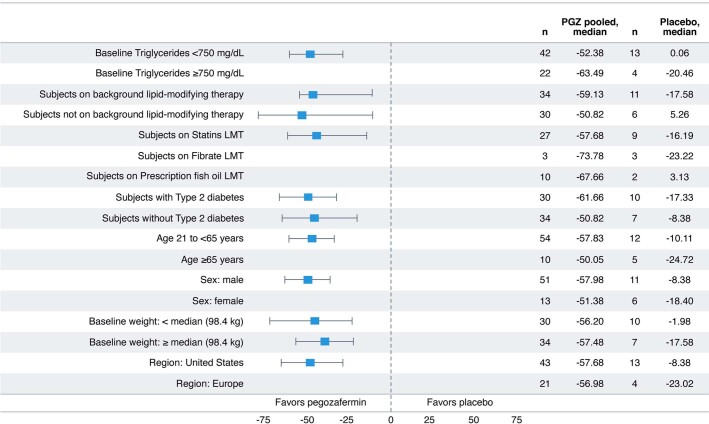
Effect of pegozafermin on serum TGs by subgroup. Shown are the 95% CIs for median differences. If the percentage of participants within a subgroup was less than 33% of the overall cohort (*n* < 6), only descriptive analysis is presented. LMT, lipid modifying therapy; PGZ, pegozafermin.

**Extended Data Fig. 3:**
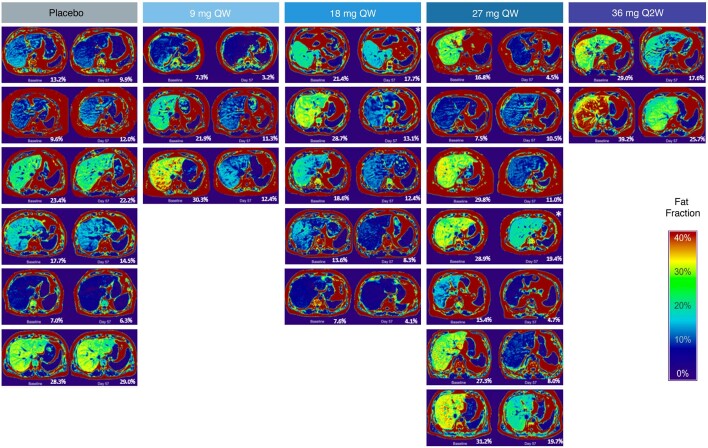
MRI-PDFF imaging. MRI-PDFF images depicting liver fat fraction at baseline and at follow-up (~day 57) in all participants with available data (23 out of 24). Images were generated using a common color-scale for all participants.

**Extended Data Fig. 4:**
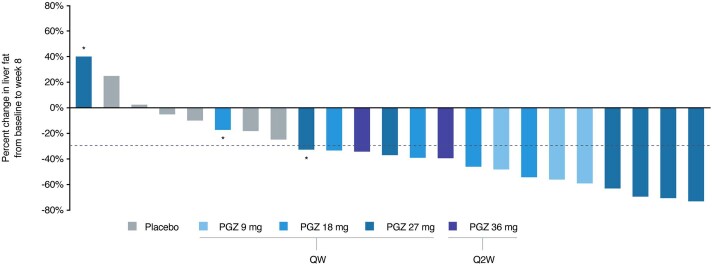
Waterfall plot. Waterfall plot showing the effect of pegozafermin on liver fat fraction assessed via MRI-PDFF. Data presented for individual participants showing relative change in liver fat from baseline to week 8. Per protocol, the week 8 follow-up MRI-PDFF assessment was to be completed within ±7 days of last dose of study drug, participants noted in asterisks (*) had follow-up MRI-PDFF performed >21 days from date of last dose. The dashed line represents a 30% reduction in liver fat which is commonly used to denote a meaningful change.

Participants were enrolled into one of the following two cohorts: (1) main study cohort (could not be on concurrent fibrate therapy) or (2) fibrate expansion cohort. The fibrate expansion cohort was initiated as a protocol amendment (v2.0) after the start of the study to evaluate pegozafermin in participants on stable fibrate therapy, as these medications are commonly used to treat SHTG. The main study cohort was randomized 1:1:1:1:1 to one of the four doses of pegozafermin (9 mg QW, 18 mg QW, 27 mg QW or 36 mg Q2W) or placebo, and the fibrate cohort was randomized 1:1 to either pegozafermin 27 mg QW or placebo QW for 8 weeks (Extended Data Fig. 1). All participants were stratified by TG level (<750 mg dl^−1^ or ≥750 mg dl^−1^ (8.5 mmol l^−1^)), with additional stratification in the main cohort by whether they were taking background therapy. An MRI-PDFF sub-study was initiated at sites able to perform MRI-PDFF imaging as participants in the fibrate expansion cohort were required to have MRI-PDFF ≥ 6.0% at enrollment. A total of 24 participants (six from the fibrate expansion cohort) received baseline MRI-PDFF measurements, of whom 23 completed a follow-up scan at the end of the study period. After completing the 8-week treatment period, all participants underwent a 4-week safety follow-up period.

Participants were required to fast for 12–14 h and abstain from alcohol for 48 h before each lipid assessment throughout the study. Following a lifestyle stabilization period (4 weeks if on stable approved LMT; up to 6 weeks if washing out ineligible LMT), a ~2-week qualification period occurred consisting of two fasting TG assessments at least 1 week apart. If mean TG levels from these two laboratory evaluations were not within the inclusion range, an additional third assessment was collected at least 1 week apart from the previous assessment. The mean TG value from the last two assessments served as TG qualification for the study and was the basis for participant TG stratification at randomization. Exclusion criteria included uncontrolled or recent diagnosis of hypertension, uncontrolled or recent diagnosis of T2DM within 6 months of screening, HbA1c ≥ 9.5%, BMI > 45 kg m^−^^2^ or cardiovascular or cerebrovascular disease.

The trial was conducted at 50 clinical sites in the United States, Hungary, Poland and the Czech Republic from September 2020 to June 2022 (Supplementary Note for a list of investigators). The trial was approved by the institutional review board or ethics committee at each site. Participants provided written informed consent. The sponsor (89bio, Inc.) designed and executed the study in collaboration with the academic authors and oversaw the clinical research organizations that performed site monitoring, data collection and analysis. All authors had access to trial data, participated in the preparation of the manuscript and had responsibility for the data and analyses.

### Study outcomes

The study objectives and endpoints were similar for the main study and fibrate cohorts. Given the small number of patients enrolled in the fibrate cohort (*n* = 6), data were pooled and presented for both cohorts. The primary efficacy endpoint was the percentage change in serum TGs from baseline to week 8. Secondary efficacy endpoints included select serum lipids and lipoproteins, metabolic markers and changes in liver fat content as assessed by MRI-PDFF. Safety endpoints included overall safety and tolerability assessments, liver function markers and immunogenicity.

Baseline TG level was defined as the average of randomization day assessment collected predose and the preceding two lipid-qualifying assessments collected during the TG qualifying period. The TG value at week 8 was defined as the average of TG values at week 7 and week 8. In case of missing TG values at week 7 or 8, the non-missing result was used as the week 8 TG value. Responder analysis of TG reduction at various threshold levels was performed, and the proportion of participants with TG normalization (<150 mg dl^−1^) was also analyzed.

### Statistical analysis

The study was designed to have at least 86% power to detect a 45% difference in TG between each of the pegozafermin arms and placebo groups, assuming 50% reduction in pegozafermin dose groups and 5% reduction in the placebo group. Both pooled pegozafermin from all dose groups and individual pegozafermin dose groups were compared with placebo. All analyses were performed at a two-sided *α* level of 0.05, without adjustment for multiplicity, and CIs were two-sided (95%). Summary descriptive statistics were used to present demographic and baseline characteristics, safety endpoints and pharmacodynamic parameters.

Efficacy analyses were conducted with the full analysis set, which included patients with at least one postbaseline TG level. Normality test was performed and if normality was severely violated, then nonparametric tests were performed. A prespecified QQ plot suggested that the distribution of TG data was highly skewed and deviated from the normality assumption required for the mixed models for repeated measures (MMRM) method. Therefore, the primary efficacy analysis was performed using a nonparametric van Elteren test, stratified by baseline TG level and background lipid therapy, to test the treatment difference using pooled data. The location shift estimate and Hodges–Lehmann two-tailed 95% CI are presented. A comparison between the individual pegozafermin dose group and placebo used the unstratified Wilcoxon rank-sum test due to low sample size. If the number of participants within any subgroup was too low for meaningful comparison (*n* < 6), only descriptive analysis was performed. Placebo-corrected change was defined as the difference in the change from baseline in a pegozafermin dose group and the change from baseline in the placebo group. Each week-8 value was defined as the average of the week-7 value, the week-8 value and any early termination values that fell within the analysis window.

Secondary efficacy endpoints were analyzed by MMRM. If the mixed model assumption was severely violated, nonparametric methods were used for the analysis. The proportion of participants with TG < 500 mg dl^−1^ at week 8 was analyzed using stratified Cochran–Mantel–Haenszel method using patients with both baseline and week 8 TG results. Unstratified chi-squared test was performed for comparisons between placebo and the individual pegozafermin dose group. Statistical analysis was performed using SAS, v9.4 or later.

### Reporting summary

Further information on research design is available in the Nature Portfolio Reporting Summary linked to this article.

## Online content

Any methods, additional references, Nature Portfolio reporting summaries, source data, extended data, supplementary information, acknowledgements, peer review information; details of author contributions and competing interests; and statements of data and code availability are available at 10.1038/s41591-023-02427-z.

### Supplementary information

Supplementary Information Supplementary Note.

Reporting Summary

## Figures and Tables

**Extended Data Table 1:**
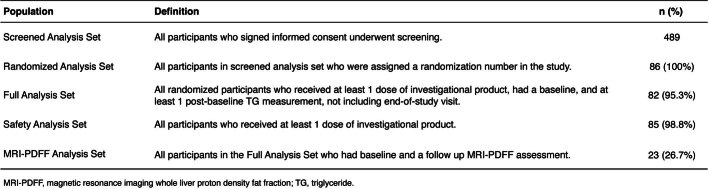
Population analysis set

**Extended Data Table 2:**
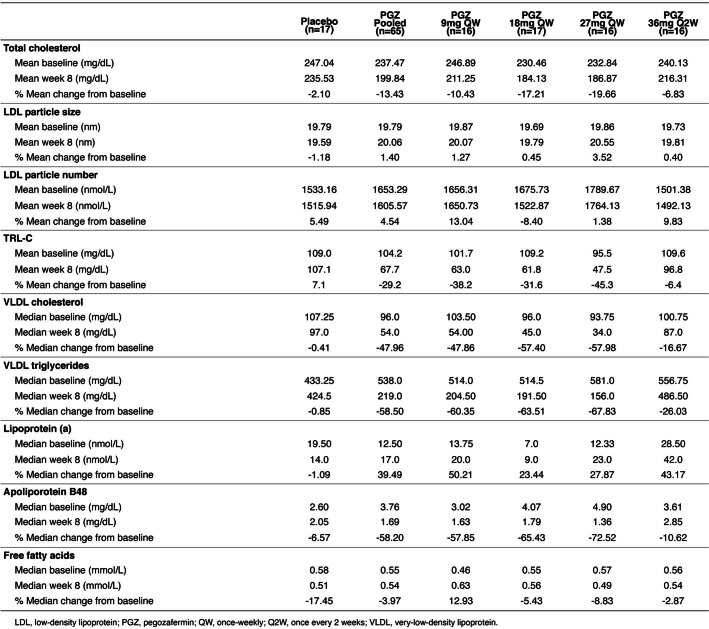
Effect of pegozafermin on serum lipids

Data are based on the full analysis set. Nonparametric methods were used for data that were highly skewed and did not meet normality assumptions.

**Extended Data Table 3:**
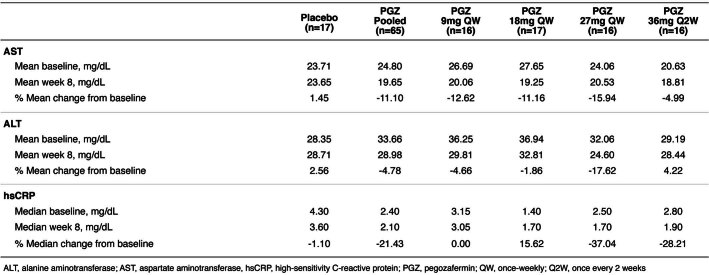
Effect of pegozafermin on markers of liver health and inflammation

Data are based on the full analysis set. Nonparametric methods were used for data that were highly skewed and did not meet normality assumptions.

## Data Availability

The datasets generated and/or analyzed during the current study are not publicly available due to proprietary considerations. Data requests pertaining to the manuscript may be made to the corresponding author and will be reviewed individually. All data will be shared in aggregate form as individual participant-level data are subject to patient privacy and prohibited from disclosure.
