# Author Correction: The FGF21 analog pegozafermin in severe hypertriglyceridemia: a randomized phase 2 trial

**DOI:** 10.1038/s41591-024-02890-2

**Published:** 2024-03-04

**Authors:** Deepak L. Bhatt, Harold E. Bays, Michael Miller, James E. Cain, Katarzyna Wasilewska, Nabil S. Andrawis, Teresa Parli, Shibao Feng, Lulu Sterling, Leo Tseng, Cynthia L. Hartsfield, Germaine D. Agollah, Hank Mansbach, John J. P. Kastelein, ENTRIGUE Principal Investigators

**Affiliations:** 1 Mount Sinai Heart, Icahn School of Medicine, Mount Sinai Health System, New York City, NY USA; 2 Louisville Metabolic and Atherosclerosis Research Center, University of Louisville School of Medicine, Louisville, KY USA; 3 Corporal Michael J. Crescenz VA Medical Center and Hospital of the University of Pennsylvania, Philadelphia, PA USA; 4 Family Medicine Clinic Science, Lampasas, TX USA; 5 ZDROWIE Osteo-Medic, Bialystok, Poland; 6 Manassas Clinical Research Center, Manassas, VA USA; 7 89bio Inc., San Francisco, CA USA; 8 Department of Vascular Medicine, Academic Medical Center, University of Amsterdam, Amsterdam, Netherlands

**Keywords:** Clinical trial design, Medical research

Correction to: *Nature Medicine* 10.1038/s41591-023-02427-z, published online 24 June 2023.

In the version of the article initially published, there were errors in Fig. 3d. The panel has now been corrected in the HTML and PDF versions of the article, and the original and revised versions can be seen below as Fig. 1.

**Fig. 1:**
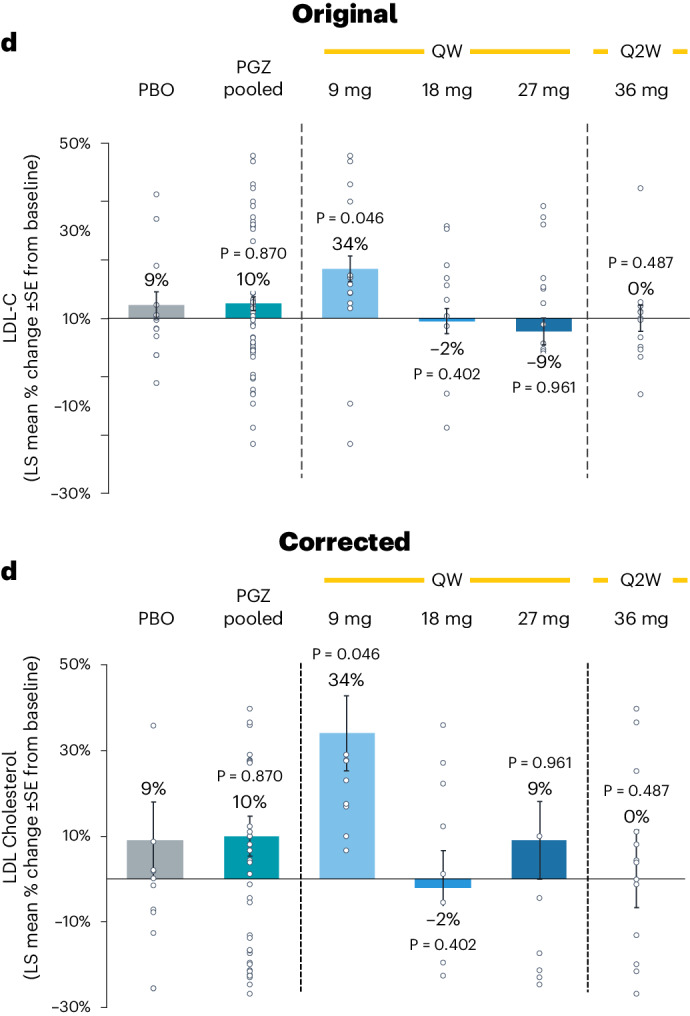
Original and revised Fig. 3d.

