# Supplementary material for: The FGF21 analog pegozafermin in severe hypertriglyceridemia: a randomized phase 2 trial
Source: Nat Med. 2023 Jun 24;29(7):1782–92. doi: 10.1038/s41591-023-02427-z (PMC10353930; doi:10.1038/s41591-023-02427-z)
Supplement: Supplementary file 1 — Supplementary Note. [file 41591_2023_2427_MOESM1_ESM.pdf]

nature medicine

Article

https://doi.org/10.1038/s41591-023-02427-z

The FGF21 analog pegozafermin in severe hypertriglyceridemia: a randomized phase 2 trial

In the format provided by the
authors and unedited

TABLE OF CONTENTS

Supplementary Note One: Investigators………………………………………………………………. 2

Supplementary Note One: ENTRIGUE Principal Investigators

Principal Investigator	Site
Robert Perry, MD*	Panax Clinical Research; Miami Lakes, FL
Douglas Denham, MD	Clinical Trials of Texas, Inc.; San Antonio, TX
Obadias Marquez, MD	ClinSearch; Chattanooga, TN
Harold Bays, MD*	L-MARC Research Center; Louisville, KY
Miguel Trevino, MD*	Innovative Research of West Florida; Clearwater, FL
Jennifer Bellucci-Jackson, MD*	Family Medicine Specialists, Inc.; Wauconda, IL
Nabil Andrawis, MD*	Manassas Clinical Research Center; Manassas, VA
Omar Benitez, MD*	South Florida Research Center, Inc.; Miami, FL
Michael Gould, MD*	Burke Primary Care; Morgantown, NC
Vanna Gold, MD*	Family Medicine Clinic Science; Lampasas, TX
James Cain, MD*	Family Medicine Clinic Science; Lampasas, TX
Bhola Rama, MD*	Rama Research, LLC; Marion, OH
Randall Huling, MD	Olive Branch Family Medical Center; Olive Branch, MS
John Lentz, MD*	Georgia Clinical Research, LLC; Lawrenceville, GA
Lucas Groban, MD	The Iowa Clinic; Des Moines, IA
Nasser Khan, MD	The Iowa Clinic; Des Moines, IA
Robert Busch, MD*	Albany Medical Center; Albany, NY
Lon Lynn, MD*	Clinical Research of West Florida, Inc.; Tampa, FL
Aslam Loya, MD	Coastal Medical Group; Houston, TX
Humberto Cruz, MD*	Florida Institute for Clinical Research; Orlando, FL
Walter Pharr, MD*	Medication Management, LLC; Greensboro, NC
Eveline Stock, MD	University of California San Francisco; San Francisco, CA

**Principal Investigator**	**Site**
Matthew Braddock, MD	Westside Center for Clinical Research; Jacksonville, FL
Colby Grossman, MD*	Palmetto Conical Research; Summerville, SC 29485
Cara East, MD*	Baylor Scott & White; Dallas, TX
Wissam Derian, MD*	Quincy Medical Group; Quincy, IL
Cynthia Brinson, MD	Central Texas Clinical Research; Austin, TX
Cindy Martinez, MD	Project 4 Research; Miami, FL
Giselle Debs-Perez, MD*	Harmony Clinical Research Center; North Miami Beach, FL
Najmuddin Karimjee, MD	DM Clinical Research/APD Clinical Research; Magnolia, TX
Venu Prabaker, MD	CareMD Associates; La Mesa, CA
Curtis Jantzi, MD	Holston Medical Group, P.C.; Kingsport, TN
Chrisette Dharma, MD	Southwest Family Medicine Associates; Dallas, TX
Jose Cevallos-Yepez, MD	Finlay Medical Research Corp.; Greenacres, FL
William Sanchez, MD*	Floridian Clinical Research; Miami Lakes, FL
Nonna Nowak, MD*	ClinicMed Daniluk, Nowak Spólka Jawna; Bialystok, Poland
Stanislaw Mazur, MD*	Centrum Medyczne Medyk; Rzeszow, Poland
Witold Zmuda, MD*	Medicome Sp. z o.o.; Oswiecim, Poland
Anna Ocicka-Kozakiewicz, MD	Nasz Lekarz Przychodnie Medyczne; Torun, Poland
Maciej Banach, MD*	Instytut Centrum Zdrowia Matki Polki; Lodz, Poland
Pawel Bogdanski, MD	Centrum Zdrowia Metabolicznego Pawel Bogdanski; Poznan, Poland
Katarzyna Wasilewska, MD*	ZDROWIE Osteo-Medic; Bialystok, Poland
Jana Cepova, MD*	Fakultni Nemocnice (FN) v Motole (Motol University Hospital); Prague, Czech Republic
Tomas Hala, MD	Center for Clinical and Basic Research (CCBR)-Synarc – Pardubice; Pardubice, Czech Republic

Principal Investigator	Site
Věra Adámková, MD*	IKEM – Institut Klinické a Experimentální Medicíny; Praha 4, Czech Republic
Marketa Hovorková, MD*	IKEM – Institut Klinické a Experimentální Medicíny; Praha 4, Czech Republic
Eva Zidkova, MD	Corintez s.r.o.; Prague, Czech Republic
Roman Gregar, MD	Clintrial s.r.o; Prague, Czech Republic
Ferenc Lakatos, MD*	Belgyogyaszati es Kardiologiai Maganrendelo; Bekescsaba, Hungary
Andrea Hornyik, MD*	Clinical Research Units Hungary; Miskolc, Borsod-Abauj-Zemplen, Hungary
Denes Pall, MD*	Debreceni Egyetem; Debrecen, Hungary
Eleonora Beke, MD*	Szent Margit Rendelointezet; Budapest, Hungary
Laszlo Konyves, MD*	Lausmed Kft.; Baja, Hungary

* Principal investigators who randomized at least one subject.
