# Supplementary material for: The FGF21 analog pegozafermin in severe hypertriglyceridemia: a randomized phase 2 trial
Source: Nat Med. 2023 Jun 24;29(7):1782–92. doi: 10.1038/s41591-023-02427-z (PMC10353930; doi:10.1038/s41591-023-02427-z)
Supplement: Supplementary file 2 — Reporting Summary [file 41591_2023_2427_MOESM2_ESM.pdf]

Corresponding author(s): *Double-blind peer review submissions: write DBPR and your manuscript number here instead of author names.*

Last updated by author(s): Jun 12, 2023

Reporting Summary

Nature Portfolio wishes to improve the reproducibility of the work that we publish. This form provides structure for consistency and transparency in reporting. For further information on Nature Portfolio policies, see our Editorial Policies and the Editorial Policy Checklist.

Statistics

For all statistical analyses, confirm that the following items are present in the figure legend, table legend, main text, or Methods section.

n/a	Confirmed	
☐	☒	The exact sample size (n) for each experimental group/condition, given as a discrete number and unit of measurement
☐	☒	A statement on whether measurements were taken from distinct samples or whether the same sample was measured repeatedly
☐	☒	The statistical test(s) used AND whether they are one- or two-sided<br>*Only common tests should be described solely by name; describe more complex techniques in the Methods section.*
☐	☒	A description of all covariates tested
☐	☒	A description of any assumptions or corrections, such as tests of normality and adjustment for multiple comparisons
☐	☒	A full description of the statistical parameters including central tendency (e.g. means) or other basic estimates (e.g. regression coefficient) AND variation (e.g. standard deviation) or associated estimates of uncertainty (e.g. confidence intervals)
☐	☒	For null hypothesis testing, the test statistic (e.g. F, t, r) with confidence intervals, effect sizes, degrees of freedom and P value noted<br>*Give P values as exact values whenever suitable.*
☒	☐	For Bayesian analysis, information on the choice of priors and Markov chain Monte Carlo settings
☒	☐	For hierarchical and complex designs, identification of the appropriate level for tests and full reporting of outcomes
☐	☒	Estimates of effect sizes (e.g. Cohen's d, Pearson's r), indicating how they were calculated

Our web collection on statistics for biologists contains articles on many of the points above.

Software and code

Policy information about availability of computer code

Data collection	RAVE EDC
Data analysis	SAS 9.4

For manuscripts utilizing custom algorithms or software that are central to the research but not yet described in published literature, software must be made available to editors and reviewers. We strongly encourage code deposition in a community repository (e.g. GitHub). See the Nature Portfolio guidelines for submitting code & software for further information.

Data

Policy information about availability of data

All manuscripts must include a data availability statement. This statement should provide the following information, where applicable:

- Accession codes, unique identifiers, or web links for publicly available datasets
- A description of any restrictions on data availability
- For clinical datasets or third party data, please ensure that the statement adheres to our policy

The datasets generated and/or analyzed during the current study are not publicly available due to proprietary considerations. Limited data may be available from the corresponding author on reasonable request.

Human research participants

Policy information about studies involving human research participants and Sex and Gender in Research.

Reporting on sex and gender

Males and females based on their biologic sex were enrolled. Due to safety concerns and need for highly effective birth control, the informed consent required agreement to utilize such methods based on ability to become or cause pregnancy. Baseline characteristics were descriptive using biologic sex, and a subset analysis was completed for the primary endpoint based on them.

Population characteristics

Male and Females (non-pregnant, non-nursing), ages 21-<=75 yrs of age

Recruitment

Participants were enrolled in 50 sites across 4 countries by Principal Investigators. Sites were routinely monitored to ensure ICH GCP guidelines were followed, including data integrity and protection of subjects' rights and safety. Site employees or their family members were not allowed to participate in the study. Subjects' participation in the study was voluntary.

Ethics oversight

The trial was conducted at 50 clinical sites in the United states, Hungary, Poland, and the Czech Republic from September 2020 to June 2022. The trial was approved by the Institutional Review Board or Ethics Committee at each site.

Note that full information on the approval of the study protocol must also be provided in the manuscript.

Field-specific reporting

Please select the one below that is the best fit for your research. If you are not sure, read the appropriate sections before making your selection.

☒ Life sciences ☐ Behavioural & social sciences ☐ Ecological, evolutionary & environmental sciences

For a reference copy of the document with all sections, see nature.com/documents/nr-reporting-summary-flat.pdf

Life sciences study design

All studies must disclose on these points even when the disclosure is negative.

Sample size

The study was designed to have at least 86% power to detect a 45% difference in TGs between each of the pegozafermin arms and placebo group, assuming 50% reduction in pegozafermin arms and 5% reduction in the placebo group. The planned sample size was 90 for the main cohort and 36 for the fibrate cohort.

Data exclusions

The efficacy analysis used the full analysis set, which included all randomized subjects who were treated, had baseline and at least one post-baseline TG value. Safety analysis included all subjects who received at least one dose of study drug.

Replication

A data management plan was put in place to ensure data integrity and data/output was QC'd by the CRO and Sponsor Biometrics groups. A statistical analysis plan outlined the statistical methodology, including procedures that would account for missing, unused, or spurious data, was finalized and approved prior to the database lock.

Randomization

The sample size was allocated equally to the placebo and each of the dose groups. The randomization was stratified by the TG level (<=750 or > 750 mg/dL) and the use of background LMT.

Blinding

The treatment assignment was blinded to the sponsor, investigator, and the patient until the final database lock completion.

Reporting for specific materials, systems and methods

We require information from authors about some types of materials, experimental systems and methods used in many studies. Here, indicate whether each material, system or method listed is relevant to your study. If you are not sure if a list item applies to your research, read the appropriate section before selecting a response.

Materials & experimental systems

n/a	Involved in the study
☒	☐ Antibodies
☒	☐ Eukaryotic cell lines
☒	☐ Palaeontology and archaeology
☒	☐ Animals and other organisms
☐	☒ Clinical data
☒	☐ Dual use research of concern

Methods

n/a	Involved in the study
☒	☐ ChIP-seq
☒	☐ Flow cytometry
☒	☐ MRI-based neuroimaging

Clinical data

Policy information about clinical studies

All manuscripts should comply with the ICMJE guidelines for publication of clinical research and a completed CONSORT checklist must be included with all submissions.

Clinical trial registration

NCT04541186

Study protocol

Included as part of manuscript submission

Data collection

The trial was conducted at 50 clinical sites in the United States, Hungary, Poland, and the Czech Republic from September 2020 to June 2022. The trial was approved by the Institutional Review Board or Ethics Committee at each site.

Outcomes

The primary efficacy endpoint of the study was percentage change in serum TG from baseline to week 8. Secondary efficacy endpoints included effect of PGZ over 8 weeks on select serum lipids and lipoproteins (i.e. proportion of subjects achieving TG<500 mg/dL; percent change in VLDL-C, LDL-C, non-HDL-C, VLDL-TG, ApoB, RLP-C and hsCRP), change in metabolic markers (fasting plasma glucose, adiponectin, and body weight), pharmacokinetics of PGZ, and change in liver fat content as assessed by MRI-PDFF

The primary efficacy analysis was planned to use a mixed model with repeated measures (MMRM), which included the treatment group, baseline TG values, background therapy use, visit, visit and treatment interactions. Because the model assumption of the MMRM method was violated, per the SAP, the non-parametric van Elteren test stratified by baseline TG level and background lipid therapy was used to test the treatment difference using pooled data. The location shift estimate, and Hodges-Lehmann 2-tailed 95% CI was presented. Secondary efficacy endpoints were analyzed via MMRM or non-parametric method similar to primary endpoint or via ANCOVA, as appropriate(e.g., for parameters that only have one post-baseline measurement, with treatment and background therapy as factors and baseline value as covariate).
